# Deubiquitinase USP5 suppresses ferroptosis in lung adenocarcinoma by deubiquitinating and stabilizing GPX3

**DOI:** 10.1002/ccs3.70121

**Published:** 2026-10-24

**Authors:** Xin Fan, Yubiao Lin, Zhigao Zhen, Yingqin Gao, Wenhui Zhen, Siyuan Zeng, Yufei Ji, Kaida Huang, Shuitu Feng

**Affiliations:** ^1^ Department of Oncology Xiamen Haicang Hospital Xiamen Fujian China; ^2^ Xiamen Cancer Hospital (The First Affiliated Hospital of Xiamen University (Haicang Campus)) Xiamen China

**Keywords:** bioinformatics, GPX3, lung adenocarcinoma, mass spectrum, ubiquitination, USP5

## Abstract

Ferroptosis is a regulated form of cell death driven by iron‐dependent lipid peroxidation and has emerged as a promising therapeutic target in nonsmall cell lung cancer (NSCLC). However, the specific mechanisms by which nonsmall cell lung cancer cells evade ferroptosis remain to be further elucidated. We conducted integrative transcriptomic analyses of published NSCLC datasets to identify ubiquitin‐specific proteases (USPs) potentially involved in the regulation of ferroptosis. Multiple USP proteins were screened and their prognostic significance evaluated, ultimately selecting USP5 for functional studies in two lung adenocarcinoma (LUAD) cell lines and patient‐derived LUAD organoids. Following treatment with the ferroptosis inducer erastin, survival rates and metastatic capacity were assessed. Ferroptosis was evaluated by measuring established ferroptotic markers and examining mitochondrial ultrastructure. Protein interactions were investigated using mass spectrometry, immunoprecipitation, and GST pull‐down assays. USP5 was significantly upregulated in LUAD tissues and correlated with poor patient prognosis. Functional experiments demonstrated that USP5 suppresses ferroptosis in LUAD cells treated with erastin, thereby promoting cell survival, proliferation, and invasiveness. Mechanistically, USP5 directly binds to and deubiquitinates GPX3, a key antioxidant enzyme, to enhance its stability. Rescue experiments confirmed that GPX3 is a functional mediator of USP5's ferroptosis‐inhibitory role. These findings are further supported in patient‐derived LUAD organoids. Our study identifies USP5 as a critical suppressor of ferroptosis in LUAD through its stabilization of GPX3, revealing a novel USP5–GPX3 regulatory axis that enables tumor resistance to oxidative stress. Targeting this pathway may represent a promising therapeutic strategy.

## Graphical abstract

1. USP5 overexpression in LUAD predicts poor prognosis, linking to tumor progression and therapy resistance. 2. USP5 deubiquitinates/stabilizes GPX3, maintaining antioxidant defense against ferroptosis. 3. Targeting inhibition of USP5 enhances LUAD ferroptosis sensitivity, offering therapeutic potential.

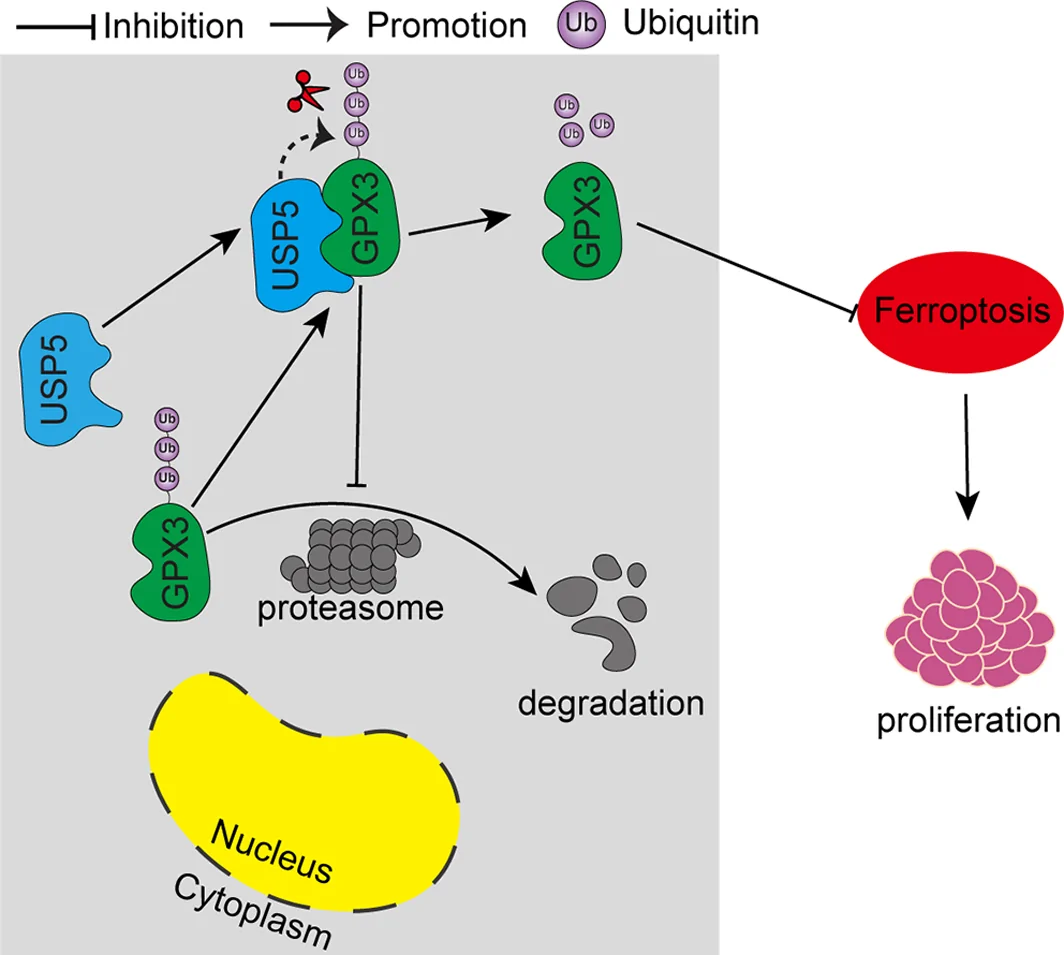

## 1 INTRODUCTION

Lung cancer is the second most commonly diagnosed cancer worldwide and remains the leading cause of cancer‐related mortality, according to the 2020 GLOBOCAN statistics.^1^ A significant proportion of patients are diagnosed at an advanced stage frequently with metastasis.^2^, ^3^ Clinically, lung cancer is classified into two main types: small‐cell lung cancer and nonsmall cell lung cancer (NSCLC).^4^ NSCLC accounts for more than 80% of all lung cancer cases, with lung adenocarcinoma (LUAD) being the most prevalent subtype. LUAD alone is responsible for nearly half of all deaths attributed to lung cancer.^5^ Despite notable advances in diagnostic and therapeutic strategies, the prognosis for LUAD remains poor. High rates of recurrence, metastasis, and therapeutic resistance continue to undermine long‐term survival.^6^, ^7^ Therefore, there is an urgent need for innovative diagnostic approaches and more effective therapeutic interventions. Achieving this goal will require a deeper and more comprehensive understanding of the molecular mechanisms that drive LUAD development and metastasis.

Ferroptosis is a distinct form of regulated cell death (RCD) characterized by iron‐dependent lipid peroxidation and oxidative damage to cellular membranes. Unlike other RCD modalities such as apoptosis, pyroptosis, or autophagy, ferroptosis is initiated by the accumulation of reactive oxygen species (ROS) and the depletion of glutathione, resulting in membrane destabilization and eventual cell death.^8^, ^9^ In recent years, the role of ferroptosis in various pathological conditions has garnered significant attention.^10^ In LUAD, ferroptosis has been implicated in multiple stages of tumorigenesis,^11^ and therapeutic strategies targeting ferroptosis have shown promise in combating NSCLC.^12^ Despite these advances, the regulatory mechanisms underlying ferroptosis in LUAD remain partially understood.

Protein ubiquitination is a critical post‐translational modification that regulates the stability, localization, and activity of numerous cellular proteins involved in diverse biological processes.^13^ This modification entails the covalent attachment of ubiquitin to substrate proteins through a sequential enzymatic cascade involving E1 activating enzymes, E2 conjugating enzymes, and E3 ligases. Importantly, ubiquitination is a reversible process mediated by deubiquitinating enzymes (DUBs), which remove ubiquitin moieties from target proteins. Among the DUBs, ubiquitin‐specific proteases (USPs) represent the largest subfamily, comprising over 50 members that participate in a wide range of physiological and pathological pathways.^14^ Increasing evidence indicates that aberrant expression or activity of USPs contributes to lung cancer development and progression. For example, USP22 enhances the motility of LUAD cells by promoting extracellular vesicle secretion,^15^ whereas USP13 drives lung squamous cell carcinoma (LUSC) by modulating lineage plasticity in lung club cells.^16^ Despite these findings, the functional roles of many USPs in lung cancer, particularly in LUAD, remain largely unexplored.

Emerging evidence has also highlighted the role of USPs and other ubiquitination‐related regulators in the modulation of ferroptosis.^17^ For instance, USP11 has been shown to inhibit ferroptosis by stabilizing key regulators such as NRF2 in liver cancer and LSH in colorectal cancer.^18^, ^19^ In contrast, USP7 facilitates ferroptosis by activating the p53/TfR1 signaling pathway in a rat model of cardiac ischemia/reperfusion injury.^20^ Nevertheless, the involvement of additional USPs in the regulation of ferroptosis and their target substrates, particularly in the context of lung cancer, remains to be characterized.

To address these questions, we conducted a bioinformatic analysis and identified several USPs overexpressed in NSCLC, negatively correlated with patient prognosis, and correlated with ferroptosis. Among them, USP5 was selected for further investigation in LUAD. In LUAD cells treated with erastin, a broadly used ferroptosis inducer, USP5 overexpression enhanced cell viability, colony formation, cell cycle progression, migration, and invasion while suppressing ferroptosis. In contrast, USP5 depletion sensitized cells to ferroptosis and impaired malignant behaviors. Mass spectrometry (MS) identified GPX3 as a potential USP5 target. Mechanistically, USP5 interacts with GPX3, reducing its ubiquitination and enhancing its stability in LUAD cells. Further function rescue experiments showed that GPX3 overexpression in USP5‐deficient LUAD cells improved malignant phenotypes and reduced ferroptosis, whereas GPX3 knockdown further amplified the effects of USP5 depletion. Finally, USP5's anti‐ferroptotic function was validated in LUAD patient‐derived tumor organoids, highlighting its potential clinical relevance.

## 2 MATERIALS AND METHODS

### 2.1 Bioinformatic analysis

Transcriptomic datasets for LUAD and LUSC from the TCGA database (https://portal.gdc.cancer.gov/) were retrieved and combined into a unified TCGA‐LUNG cohort. An additional NSCLC dataset, GSE101929, was obtained from the GEO database (https://www.ncbi.nlm.nih.gov/geo/). Differential gene expression analysis was performed using the “limma” R package (v3.56.2) in R (v4.3.0), with a threshold of log2FC > 0 and FDR <0.05. USP expression levels between tumor and adjacent normal tissues were compared using unpaired Student's *t*‐tests and visualized with the “ggplot2” package (v3.5.1). Venn diagrams were generated via the Venny platform (https://bioinfogp.cnb.csic.es/tools/venny/).

The association between individual USP expression and patient survival was initially assessed using KM plotter (https://www.kmplot.com/) and validated in the TCGA‐LUNG cohort through Kaplan–Meier (KM) survival analysis using the “survival” R package (v3.5–7). Optimal expression cutpoints were determined using the “survminer” package (v0.4.9) based on log2(TPM+1) values. Corresponding clinical data were obtained from UCSC Xena (https://xena.ucsc.edu/).

Gene Ontology (GO) and Kyoto Encyclopedia of Genes and Genomes (KEGG) enrichment analyses were performed on differentially expressed genes between USP5‐high and USP5‐low groups (based on median USP5 mRNA levels) using the “GSEA” R package (v4.3.3). The top 20 enriched GO terms or pathways were visualized with “ggplot2.” Pearson correlations between USP expression and ferroptosis‐related genes were calculated using the “stats” package (v4.3.0) with the cor.test function, and results were plotted using the “graphics” package (v4.3.0).

### 2.2 Cell culture

A549 (IM‐H113) and HCC827 (IM‐H246) cell lines were obtained from IMMOCELL Biotechnology (China). The HEK293T cell line is maintained by our laboratory. A549 and HEK293T cells were cultured in DMEM (IMC‐305, IMMOCELL) supplemented with 10% fetal bovine serum (FBS; FCS500, ExCell Bio, China) and 1× penicillin‐streptomycin (15070063, IMMOCELL). HCC827 cells were maintained in a specialized medium (IM‐H246‐1, IMMOCELL) as recommended. All cells were cultured at 37°C in a humidified incubator with 5% CO_2_ and 95% air and were routinely tested for *Mycoplasma* contamination using the PlasmoTest kit (rep‐pt1, InvivoGen, USA).

### 2.3 Plasmid construction

For gene knockdown, three short hairpin RNAs (shRNAs) targeting USP5 (shUSP5) or GPX3 (shGPX3), along with a nontargeting control (shNC), were designed and cloned into the pLKO.1 lentiviral vector. Lentivirus production was outsourced to Anti‐hela Biological Technology (China). For overexpression studies, the USP5 coding sequence was cloned into the pLVX‐CMV‐HA‐pGK‐puro vector to generate the HA‐USP5 construct. Coding sequences for NCOA4, ATG16L1, HMOX1, and GPX3 were inserted into the pcDNA3.1‐FLAG vector to create the FLAG‐tagged constructs such as FLAG‐NCOA4, FLAG‐ATG16L1, FLAG‐HMOX1, and FLAG‐GPX3, respectively. To overexpress ubiquitin, its coding sequence was cloned into the pcDNA3.1‐His vector, generating the His‐Ub construct. All plasmids were sequence‐verified to confirm accuracy. Primer sequences used for plasmid construction are listed in Supporting Information S1: Table S1.

### 2.4 Stable cell line establishment

LUAD cells were infected with lentiviral particles at an optimized multiplicity of infection (MOI) of 15. Stable cell lines were generated by culturing infected cells in a medium containing 2 μg/mL puromycin (60210ES25, Yeason, China) and 10 μg/mL polybrene (40804ES76, Yeason). After 48 hours, the medium was replaced with fresh medium containing puromycin alone. The medium was refreshed every 3 days. Following 7 days of selection, surviving cells were transferred to a puromycin‐free medium and collected for subsequent experiments.

### 2.5 Quantitative PCR

Total RNA was extracted using the RaPure Total RNA Kit (R4011‐02, Magen, China), and RNA quality was assessed with a Nanodrop spectrophotometer (ND‐8000‐GL, Thermo Fisher Scientific, USA). Reverse transcription was carried out using the All‐in‐One First‐Strand SuperMix with dsDNase (EG15133S, ny‐bio, China). qPCR was performed on a QuantStudio 3 system (A28573, Thermo Fisher Scientific) using ChamQ SYBR Color qPCR Master Mix (Q431‐02, Vazyme, China). Each group included biological and technical triplicates. Relative RNA expression levels were calculated using the 2^−△△Ct^ method. Primers are as follows: qUSP5‐F: 5′‐ GAGCTCGACATCTCCCAGTT‐3′, qUSP5‐R: 5′‐CGATGTCGGAGAGGAGAAGTG‐3’; qGPX3‐F: 5′‐AGAGCCGGGGACAAGAGAA‐3′, qGPX3‐R: 5′‐ATTTGCCAGCATACTGCTTGA‐3’; q18S‐F: 5′‐CGACGACCCATTCGAACGTCT‐3′, q18S‐R: 5′‐CTCTCCGGAATCGAA CCCTGA‐3’.

### 2.6 Western blot

Proteins were extracted using RIPA lysis buffer (20–188, Sigma‐Aldrich, USA) supplemented with 1 mM phenylmethylsulfonyl fluoride (PMSF; 10837091001, Sigma‐Aldrich), following the manufacturer's instructions. Protein concentration was measured using a BCA Protein Assay Kit (23225, Thermo Fisher Scientific). Samples were mixed with loading buffer and denatured at 95°C for 5 min. Proteins were separated by SDS‐PAGE and transferred to PVDF membranes, which were then blocked with 4% nonfat milk (Beyotime Biotechnology, China) and incubated overnight at 4°C with primary antibodies. After washing, membranes were incubated with HRP‐conjugated secondary antibodies for 2 h at room temperature. Following additional washes with TBST (TBS + 0.1% Tween‐20), signals were visualized using ECL reagent (Beyotime Biotechnology) and exposed to X‐ray films. Protein band intensities were quantified using ImageJ software (NIH, USA), normalized to internal controls. All experiments were performed in triplicate for statistical analysis. For details of the Western blot antibodies, please refer to Supporting Information S1: Table S2.

### 2.7 Cell viability and IC_50_ assay

Cell viability was evaluated using the MTT assay (ST316, Beyotime, China). LUAD cells were seeded in 96‐well plates at a density of 1 × 10^4^ cells per well and incubated for the indicated durations. Subsequently, 20 μL of MTT solution (5 mg/mL) was added to each well, followed by incubation at 37°C for 4 h. The medium was then removed, and 150 μL of DMSO was added to dissolve the formazan crystals, with gentle shaking for 10 min at room temperature. Optical density at 490 nm (OD_490_) was measured using a microplate reader (A51119500C, Thermo Fisher Scientific). To determine the IC50 values for Erastin (Era) treatment, cells were treated with different concentrations of Era (2⁻⁵, 2⁻³, 2⁻¹, 2¹, 2³, 2⁻⁵, and 2⁷) for 24 hours. After incubation with CCK8 (40203ES76, Yeasen, China) solution, the OD values were measured (OD450). Cell viability (%) = (OD_treatment_‐OD_blank_)/(OD_control_‐OD_blank_) x 100%. The IC50 was then obtained using linear fitting. The calculation formula is: y = Bottom + (Top‐Bottom) / (1 + 10^(LogIC50‐X)^ ^x HillSlope^). Here, Y represents cell viability (%), X represents log10 (treatment concentration), Top is the upper limit of the plateau, Bottom is the lower limit of the plateau, and HillSlope is the slope (Hill coefficient). Each condition was assayed in technical triplicate, and experiments were repeated three times independently.

### 2.8 Colony formation assay

LUAD cells were seeded in 6‐well plates at approximately 700 cells per well and cultured for 12 days to allow colony formation. After incubation, the medium was removed, and cells were fixed with 4% paraformaldehyde for 30 min at room temperature, followed by staining with 0.5% crystal violet for 15 min. The plates were then washed, imaged, and the number of colonies was quantified using ImageJ software. Each condition was tested in three independent experiments.

### 2.9 Cell cycle analysis

For cell cycle evaluation, 5 × 10^6^ LUAD cells were collected and fixed in 70% ethanol overnight at −20°C. Cells were then washed with PBS and incubated with 20 μL RNase A at 37°C for 0.5 h. After RNase A treatment, cells were stained with 5 μL 7‐AAD solution (ORCCY014A, Aoruicell, China) in PBS and incubated in the dark at 4°C for 0.5 h. Samples were analyzed by flow cytometry using a NovoCyte 1300 instrument (ACEA, USA). The data were analyzed with the accessory software.

### 2.10 BODIPY 581/591 C11 staining

Lipid peroxidation was assessed by flow cytometry using BODIPY 581/591 C11 (D3861, Invitrogen). LUAD cells were seeded in 12‐well plates (triplicate) and cultured for 24 h, followed by treatment with test compounds for the indicated duration. Cells were then incubated with 2 μM BODIPY 581/591 C11 in a fresh medium at 37°C for 20 min. After trypsinization and PBS washing, fluorescence intensity was acquired on a NovoCyte 1300 flow cytometer (ACEA, USA). Data were analyzed using FlowJo (BD Biosciences), and mean fluorescence intensity (MFI) was normalized to the untreated control.

### 2.11 Transwell assay

Cell migration and invasion assays were performed using 24‐well plates with 8 μm pore Transwell chambers (3422, Corning, USA). For invasion assays, each upper chamber was pre‐coated with 100 μL of 1:5 diluted Matrigel (356235, BD Biosciences, USA) and incubated at 37°C for 2 hours. LUAD cells were digested, washed, and resuspended in a serum‐free medium at a concentration of 3 × 10^5^ cells/mL. A 200‐μL cell suspension was then added to the upper chamber, whereas 700 μL of complete medium was placed in the lower chamber. After 48 hours of incubation, cells that migrated or invaded through the membrane were fixed with 4% paraformaldehyde for 30 min, stained with 0.05% crystal violet for 5 min, and imaged using an inverted microscope (IX73, OLYMPUS, Japan). ImageJ software was used for quantification.

### 2.12 Evaluations of malondialdehyde, glutathione, and Fe^2+^ levels

Lipid peroxidation, indicated by MDA levels, was measured using an MDA assay kit (BC0020, Solarbio) following the manufacturer's instructions. LUAD cells or organoids were lysed, and lysates were incubated with MDA reagent and heated at 95°C for 40 min to form MDA–TBA adducts. OD_532_ was measured, and MDA concentrations were calculated using a standard curve.

Reduced (GSH) and oxidized (GSSG) glutathione levels were measured using a GSH/GSSG assay kit (S0053, Beyotime, China). After lysis of LUAD cells or organoids, the supernatants were collected for analysis. For GSH detection, chromogenic reagent and assay buffer were added, and absorbance at OD_412_ was recorded. For GSSG measurement, supernatants were pre‐treated with a derivatizing agent. GSH and GSSG concentrations were determined using standard curves, and the GSH/GSSG ratio was calculated accordingly.

Fe^2+^ levels were assessed using an iron colorimetric assay kit (E‐BC‐K881‐M, Elabscience, USA). Cells or organoids were lysed with the provided buffer, and supernatants were obtained by centrifugation. An iron probe was added to the supernatant to form a colored complex with Fe^2+^, and OD_593_ was measured after 10 min of incubation at 37°C. Fe^2+^ concentrations were calculated from a standard curve and normalized to total protein content, measured by BCA assay.

### 2.13 DCF‐DA staining

ROS levels were detected by DCF‐DA staining. LUAD cells (∼1.0 × 10^7^ cells/mL) were incubated with 10 μM DCF‐DA (50101ES01, Yeasen, China) in PBS for 15 min in the dark, washed with PBS, then digested, and analyzed by flow cytometry. Fluorescence intensity was used to quantify ROS levels.

### 2.14 Co‐immunoprecipitation assays

Co‐IP was performed by co‐transfecting 5 μg of plasmids into 5 × 10^6^ LUAD cells seeded in 6‐well plates. After 48 h, cells were harvested and lysed in RIPA buffer. Lysates were incubated with Protein G Dynabeads (10003D, Thermo Fisher Scientific) pre‐bound to either anti‐HA (Proteintech, 66006‐2‐Ig) or anti‐FLAG (Proteintech, 66008‐4‐Ig) antibodies at 4°C for 2 h. After three washes with RIPA buffer, the immunoprecipitates were eluted, denatured, and analyzed by Western blotting.

### 2.15 GST pull‐down

His‐tagged GPX3 (His‐GPX3), GST‐tagged USP5 (GST‐USP5), and GST proteins were generated by Anti‐hela Biological Technology. Equal amounts of His‐GPX3 were incubated with either GST‐USP5 or GST control protein. An aliquot of each mixture was denatured and centrifuged to serve as input controls. The remaining mixtures were incubated with 20 μL of Glutathione Sepharose 4B beads (17075601, Sigma‐Aldrich) at 4°C for 6 h with gentle rotation. Afterward, the beads were collected by centrifugation and washed five times with ice‐cold PBS to remove unbound proteins. Bound proteins were eluted in 80 μL of elution buffer and denatured, followed by centrifugation. The resulting supernatants were collected and analyzed by Western blotting.

### 2.16 Mass spectrometry

LUAD cells were seeded into 6‐cm plates and cultured to approximately 75% confluence. Cells were then transfected with either the empty vector or HA‐USP5 plasmids and cultured for an additional 24 h. Afterward, cells were rinsed twice with ice‐cold PBS and lysed using the lysis buffer provided in the HA‐tag IP Kit (P2185M, Beyotime) with homogenization on ice. Lysates were centrifuged at 13,000 g for 15 min at 4°C, and the resulting supernatants were subjected to IP following the manufacturer's protocol. Eluted proteins were denatured, separated by SDS‐PAGE, and processed for silver staining. The corresponding gel bands were analyzed by MS using a timsTOF Pro instrument (Bruker, Germany).

### 2.17 Transmission electron microscopy

LUAD cells or organoids were initially fixed in 2.5% glutaraldehyde, then digested, pelleted, and resuspended for an additional 2‐h fixation in 2.5% glutaraldehyde at room temperature. The samples were subsequently processed for TEM by Pinuofei Biotechnology (China) and imaged using a Hitachi HT7500 instrument.

### 2.18 Organoid establishment and culture

LUAD tissue samples were collected using sterile forceps and placed in a cell culture dish containing ice‐cold PBS supplemented with 1% antibiotics. The tissues were then washed several times in cold PBS with 1% antibiotics and minced into ∼1 mm^3^ fragments using sterile scissors. Tissue fragments were further washed until the supernatant became clear. The washed tissue was resuspended in digestive buffer and incubated at 37°C with gentle mixing every 10 min. After digestion, samples were centrifuged at 400 g for 5 min, and the supernatant was discarded. The resulting cell pellet was resuspended in 0.1% BSA in cold PBS and filtered through a 70‐μm cell strainer. Red blood cells were lysed by resuspending the filtrate in 2–3 mL of RBC lysis buffer, incubating at room temperature for 2–3 min, and then quenching with PBS. After centrifugation at 400 g for 5 min, the supernatant was discarded. The resulting cell pellet was resuspended in Matrigel, and 40 μL of the suspension was plated into each well of a 24‐well plate. The plate was incubated at 37°C with 5% CO_2_ for 20 min to allow Matrigel solidification. Pre‐warmed organoid culture medium (IMV‐T18, IMMOCELL) supplemented with 10 μM Y‐27632 (HY‐10071, MedChemExpress, USA) was then added. The medium was refreshed every 3 days to maintain optimal growth conditions. All experiments were approved by the Medical Ethics Committee, and written consent was obtained from patients.

### 2.19 Immunofluorescence and hematoxylin and eosin (H&E) staining

Immunofluorescence: LUAD organoids were paraffin‐embedded and sectioned at 5 μm thickness. Sections were dewaxed using xylene and rehydrated through a graded ethanol series into water. Antigen retrieval was performed by immersing the slides in boiling EDTA buffer (pH 9.0) and incubating them in a 90°C water bath for 0.5 h. After cooling to room temperature naturally and washing in distilled water 3 times, excess moisture was removed, and a hydrophobic barrier was drawn around each tissue section using a PAP pen. Nonspecific binding was blocked with 5% BSA for 10 min, followed by three additional PBS washes. Sections were then incubated with primary antibodies at 4°C overnight. After washing three times with PBS, secondary antibodies were applied and incubated at room temperature for 1 h. Slides were washed again and mounted with an anti‐fade mounting medium. Imaging was performed using an LSM 900 confocal microscope (Zeiss, Germany). The following antibodies were used: Ki‐67 antibody (9027, Cell Signaling Technology, USA, 1:500), Napsin A antibody (A124485, Abclonal, 1:500), TTF1 antibody (A3292, Abclonal, 1:500), and CoraLite488‐conjugated Goat Anti‐Rabbit IgG (SA00013‐2, Proteintech, 1:800). Signal quantification (Integrated Density, IntDen or % positive cells) was conducted by Image J.

HE: Paraffin sections of LUAD organoids were dewaxed in eco‐friendly dewaxing solutions I and II (Servicebio, G1128, 15 min each), rehydrated via graded ethanol, and stained with hematoxylin (Servicebio H&E kit, G1101, 2–5 min). After bluing and tap water rinsing, sections were counterstained with eosin (15–30 s). Slides were dehydrated in graded ethanol, cleared in dewaxing solutions I and II (2 min each), mounted with neutral gum (China National Pharmaceutical Group, G1076), and imaged with an LSM 900 confocal microscope (Zeiss, Germany).

### 2.20 Statistical analysis

Data analysis and visualization were performed using R or GraphPad Prism (version 8, GraphPad Software, USA). Results are presented as mean ± standard deviation (SD). In bar‐dot plots, each data point represents either a biological replicate and/or the mean of technical triplicates. Specific statistical tests used are detailed in the corresponding figure legends. A *p*‐value of <0.05 was considered statistically significant.

## 3 RESULTS

### 3.1 Screening of oncogenic USPs potentially involved in the regulation of ferroptosis in NSCLC

To explore which USP family members may possess oncogenic properties and participate in ferroptosis regulation in NSCLC, we analyzed transcriptomic profiles from the TCGA‐LUNG and GSE101929 datasets. Nine USPs were found to be significantly upregulated in NSCLC tumor tissues (Figure 1A). Among these, USP5, USP14, USP18, USP28, and USP39 were consistently overexpressed in tumors compared with adjacent normal lung tissues (Figure 1B). Further stratification analyses revealed that none of these USPs exhibited differential expression across various age groups. Notably, USP5, USP14, and USP28 showed higher expression in male patients, whereas USP5, USP14, and USP39 were upregulated in deceased patients. These USPs also demonstrated partial expression differences among tumor stages, possibly due to limited sample size (Supporting Information S1: Figure S1A–E).

**FIGURE 1:**
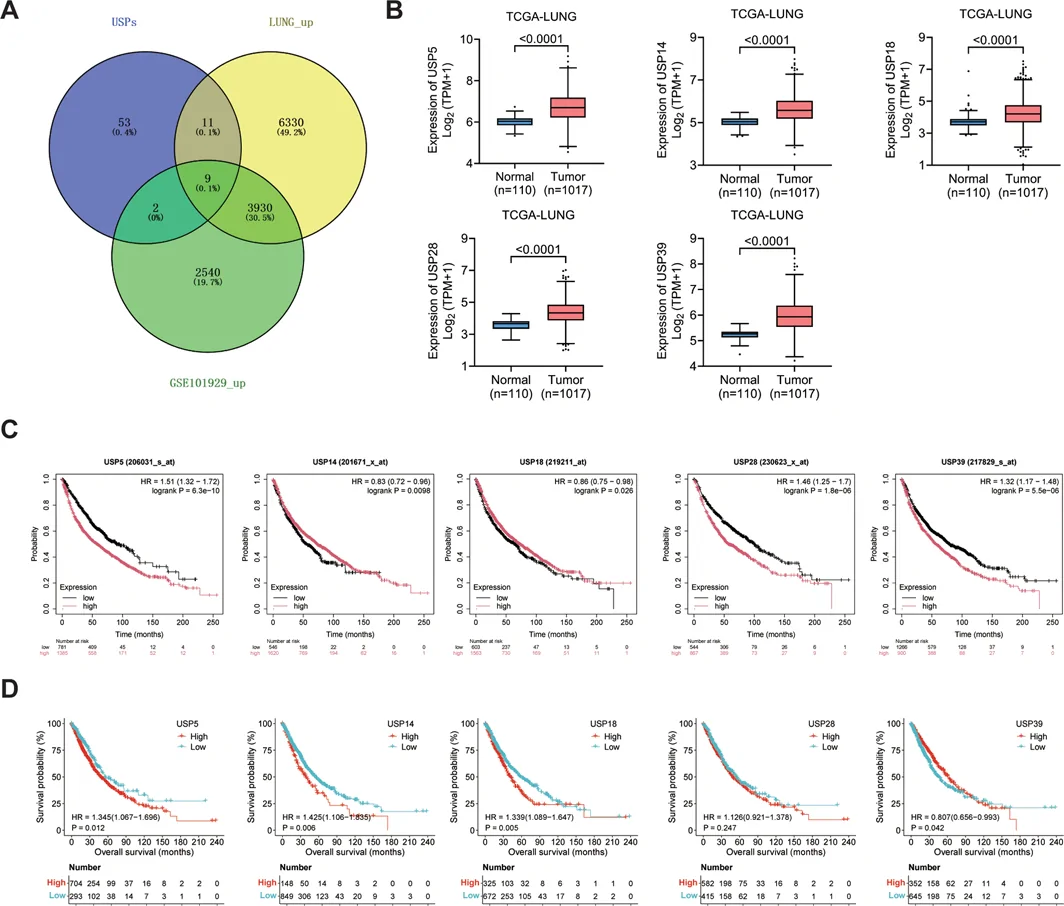
Identification of oncogenic USPs potentially regulating ferroptosis in NSCLC. (A) Venn diagram showing overlap of overexpressed USPs in NSCLC across TCGA‐LUNG and GSE101929 datasets. (B) Boxplots showing mRNA expression levels of USP5, USP14, USP18, USP28, and USP39 in tumor versus adjacent normal tissues in the TCGA‐LUNG dataset. (C) Kaplan–Meier survival curves from KM plotter showing associations of individual USP expression with overall survival in NSCLC patients. (D) Kaplan–Meier survival analysis of TCGA‐LUNG cohort demonstrating individual USP expression with overall survival in NSCLC patients. NSCLC, nonsmall cell lung cancer; USPs, ubiquitin‐specific proteases.

To investigate the prognostic implications of these USPs, we conducted survival analyses using KM plotter. Results showed that high expression of USP5, USP28, and USP39 correlated with shorter patient survival, whereas elevated USP14 and USP18 were associated with better prognosis (Figure 1C). Validation using the TCGA‐LUNG cohort indicated that high expression of USP5, USP14, and USP18 predicted worse outcomes, whereas USP28 and USP39 were not significantly or marginally correlated with prognosis (Figure 1D). It is notable that we employed different cohorts for our analysis, and the results may vary due to differences in subtypes; however, across all study cohorts, only USP5 consistently demonstrated prognostic significance. Functional enrichment analysis of USP5‐associated differentially expressed genes revealed significant enrichment in cell division and DNA replication‐related GO terms (molecular function, MF; cell component, CC; biological process, BP) and KEGG pathways (Supporting Information S1: Figure S2A). Additionally, USP5 expression was positively correlated with several ferroptosis‐related genes, including GPX4, NRF2, RGS4, and SLC7A11, based on Pearson correlation coefficients (Supporting Information S1: Figure S2B). These findings highlight USP5 as a potential oncogenic regulator of ferroptosis in NSCLC.

### 3.2 USP5 inhibits the ferroptosis of LUAD cells exposed to erastin

To further dissect the role of USP5 in the ferroptosis of NSCLC, we utilized two LUAD cell lines, A549 and HCC827, as LUAD represents the predominant NSCLC subtype. Ferroptosis was induced using erastin, and CCK8 assays determined the IC50 values to be 20.4 μM for A549 and 36 μM for HCC827 cells (Supporting Information S1: Figure S3A). These concentrations were used for subsequent experiments. Overexpression of USP5 significantly enhanced the viability of erastin‐treated A549 and HCC827 cells, whereas overexpression of USP14, USP18, or USP39 had minimal or opposite effects (Supporting Information S1: Figure S3B).

We developed three USP5‐targeting shRNAs and USP5 overexpression constructs (USP5‐OE) and then confirmed their efficacy in HEK293T cells via qPCR analysis (Supporting Information S1: Figure S3C). Stable USP5‐deficient and overexpressing LUAD cell lines were generated, and knockdown/overexpression was confirmed by qPCR (Supporting Information S1: Figure S3D). Erastin treatment did not notably alter USP5 mRNA expression. Subsequent MTT assays showed that USP5 knockdown reduced A549 cell viability, while USP5 overexpression enhanced HCC827 cell viability (Figure 2A). Colony formation assays yielded consistent results, showing reduced colony numbers upon USP5 knockdown and increased colony formation following USP5 overexpression (Figure 2B). Cell cycle analysis by 7‐AAD staining revealed G0/G1 arrest in USP5‐depleted A549 cells, whereas USP5 overexpression promoted HCC827 cell cycle progression (Figure 2C,D). Transwell assays showed impaired migration and invasion in USP5‐depleted A549 cells, whereas USP5 overexpression promoted these processes in HCC827 cells (Figure 2E–H).

**FIGURE 2:**
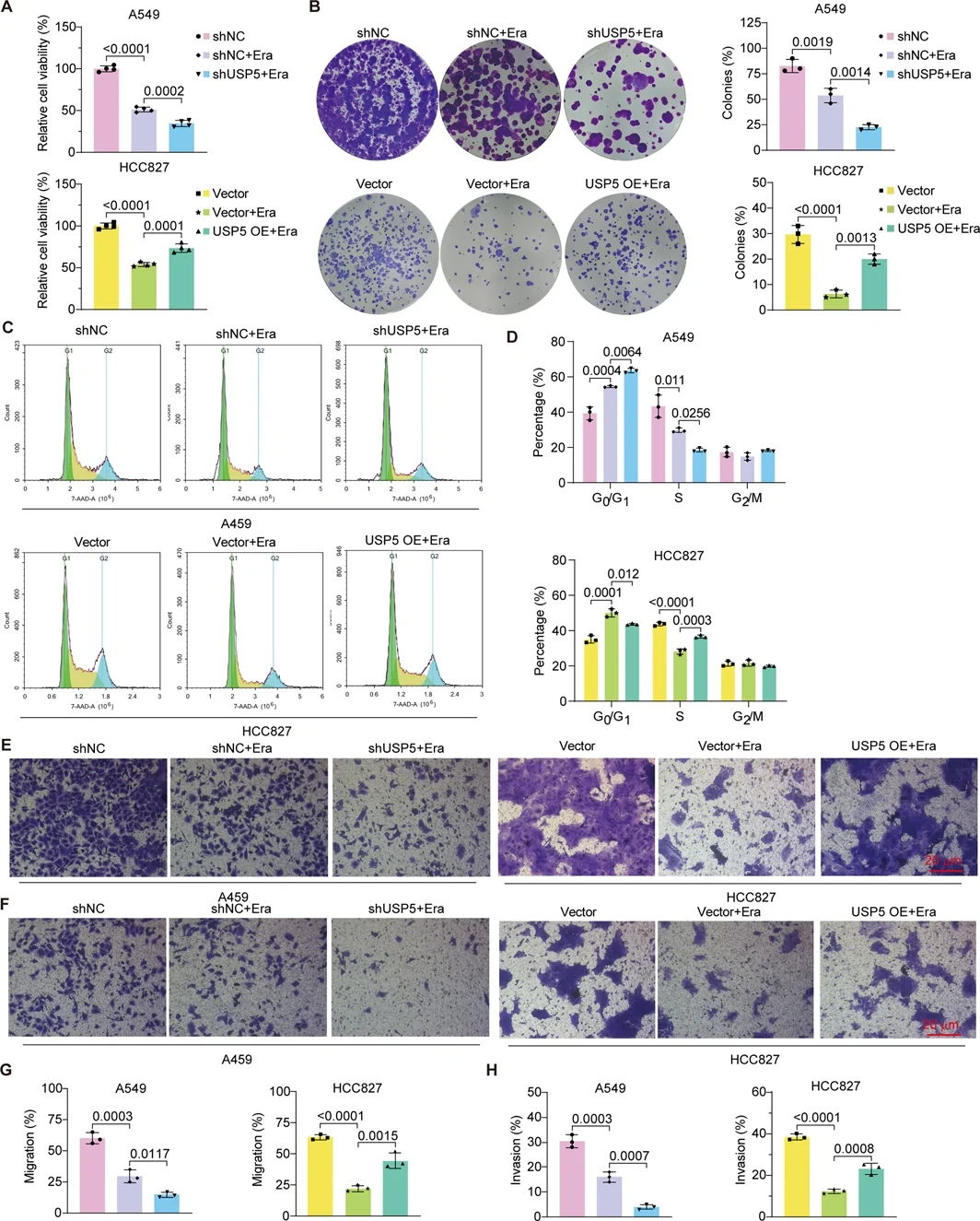
USP5 promotes malignancy in erastin‐treated LUAD cells. (A) MTT assays showing reduced viability of A549 cells upon USP5 knockdown and increased viability of HCC827 cells upon USP5 overexpression following erastin treatment. (B) Colony formation assays demonstrating impaired clonogenicity with USP5 depletion and enhanced colony formation with USP5 overexpression under ferroptotic stress. (C) Cell cycle analysis via 7‐AAD staining showing G_0_/G_1_ arrest in USP5‐deficient A549 cells and S phase progression in USP5‐overexpressing HCC827 cells. (D) Statistical analysis of Figure C. (E, F) Transwell migration and invasion assays indicating impaired motility upon USP5 knockdown and enhanced invasiveness upon overexpression. (G, H) Statistical analysis of Figures E (Migration) and F (Invasion). Era, erastin. Statistical significance was assessed using the one‐way ANOVA followed by Tukey's HSD test. HSD, honest significant difference.

Biochemical analyses revealed elevated MDA, GSSG, and Fe^2+^ levels, along with decreased GSH levels, in erastin‐treated A549 cells following USP5 knockdown. Conversely, USP5 overexpression in HCC827 cells led to reduced MDA, GSSG, and Fe^2+^ levels, and increased GSH content (Figure 3A–E). DCF‐DA staining confirmed increased ROS production in USP5‐deficient A549 cells and decreased ROS levels in USP5‐overexpressing HCC827 cells (Figure 3F). BODIPY 581/591 C11 staining revealed increased lipid peroxidation—a hallmark of ferroptosis—in USP5‐deficient A549 cells, whereas lipid peroxidation was reduced in USP5‐overexpressing HCC827 cells (Figure 3G). TEM showed that USP5 knockdown aggravated mitochondrial damage in A549 cells, whereas USP5 overexpression preserved mitochondrial integrity in HCC827 cells (Figure 3H). Western blot analysis demonstrated that USP5 knockdown reduced, and overexpression increased, the expression of ferroptosis inhibitors such as SLC7A11, GPX4, and FTH1 (Figure 3I). Notably, erastin exhibited no effect on USP5 protein levels in A549 and HCC827 cells. These findings collectively establish USP5 as a ferroptosis suppressor in LUAD.

**FIGURE 3:**
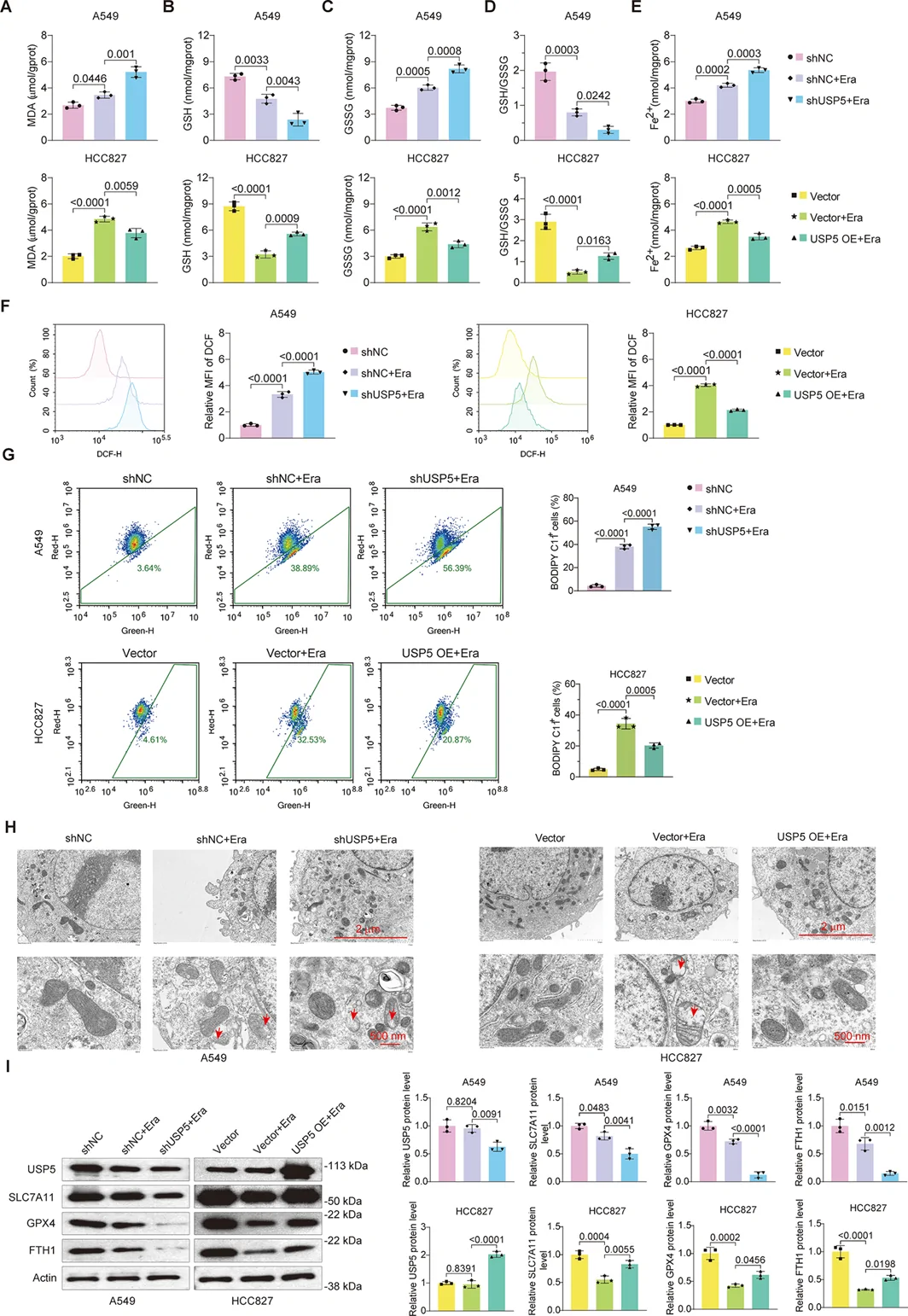
USP5 modulates biochemical and molecular markers of ferroptosis in LUAD cells. (A‐E) Quantification of MDA, GSSG, GSH, and Fe2+ levels in A549 and HCC827 cells with USP5 knockdown or overexpression following erastin treatment. (F) DCF‐DA staining showing increased ROS in USP5‐deficient A549 cells and decreased ROS in USP5‐overexpressing HCC827 cells with erastin treatment. (G) BODIPY 581/591 C11 staining results showed that cellular lipid peroxidation was increased in cells with USP5 knockdown, whereas it was reduced in cells with USP5 overexpression. (H) TEM images showing mitochondrial damage exacerbation with USP5 depletion and alleviation with overexpression. Red arrows indicated damaged mitochondria. (I) Western blot analysis of USP5 and ferroptosis‐related proteins (SLC7A11, GPX4, and FTH1) in LUAD cells, with or without USP5 modulation and erastin treatment. *p*‐values were calculated using the one‐way ANOVA followed by Tukey's HSD test. ROS, reactive oxygen species.

### 3.3 USP5 stabilizes GPX3 by removing its ubiquitination in LUAD cells

To elucidate the molecular mechanism by which USP5 inhibits ferroptosis, we conducted IP‐MS in LUAD cells overexpressing HA‐tagged USP5 (Table S3). Among the interacting proteins identified, several were ferroptosis‐related, including NCOA4, ATG16L1, HMOX1, and GPX3.^21^, ^22^, ^23^, ^24^ Co‐IP assays confirmed a specific interaction between USP5 and GPX3 in A549 cells (Figure 4A). To determine whether GPX3's degradation is mediated by the ubiquitin‐proteasome system, we treated A549 and HCC827 cells with protein synthesis inhibitor CHX and varying doses of proteasomal inhibitor MG132. Western blotting showed that GPX3 degradation was attenuated in a dose‐dependent manner by MG132, suggesting proteasomal regulation (Figure 4B). Further co‐IP and GST‐pull down assays revealed a direct binding between USP5 and GPX3 (Figure 4C,D). Since USP5 is a deubiquitinase, we evaluated the influence of USP5 on the ubiquitination of GPX3 in A549 and HCC827 cells. IP assays revealed that USP5 depletion increased GPX3 ubiquitination in A549 cells, whereas overexpression reduced it in HCC827 cells (Figure 4E). Moreover, GST‐USP5 pulled down GPX3 had a markedly lower ubiquitination level than that precipitated by GST control (Figure 4F). CHX chase assays further confirmed that USP5 stabilized GPX3 protein levels over time (Figure 4G). These results establish that USP5 directly binds to and deubiquitinates GPX3, thereby stabilizing it in LUAD cells.

**FIGURE 4:**
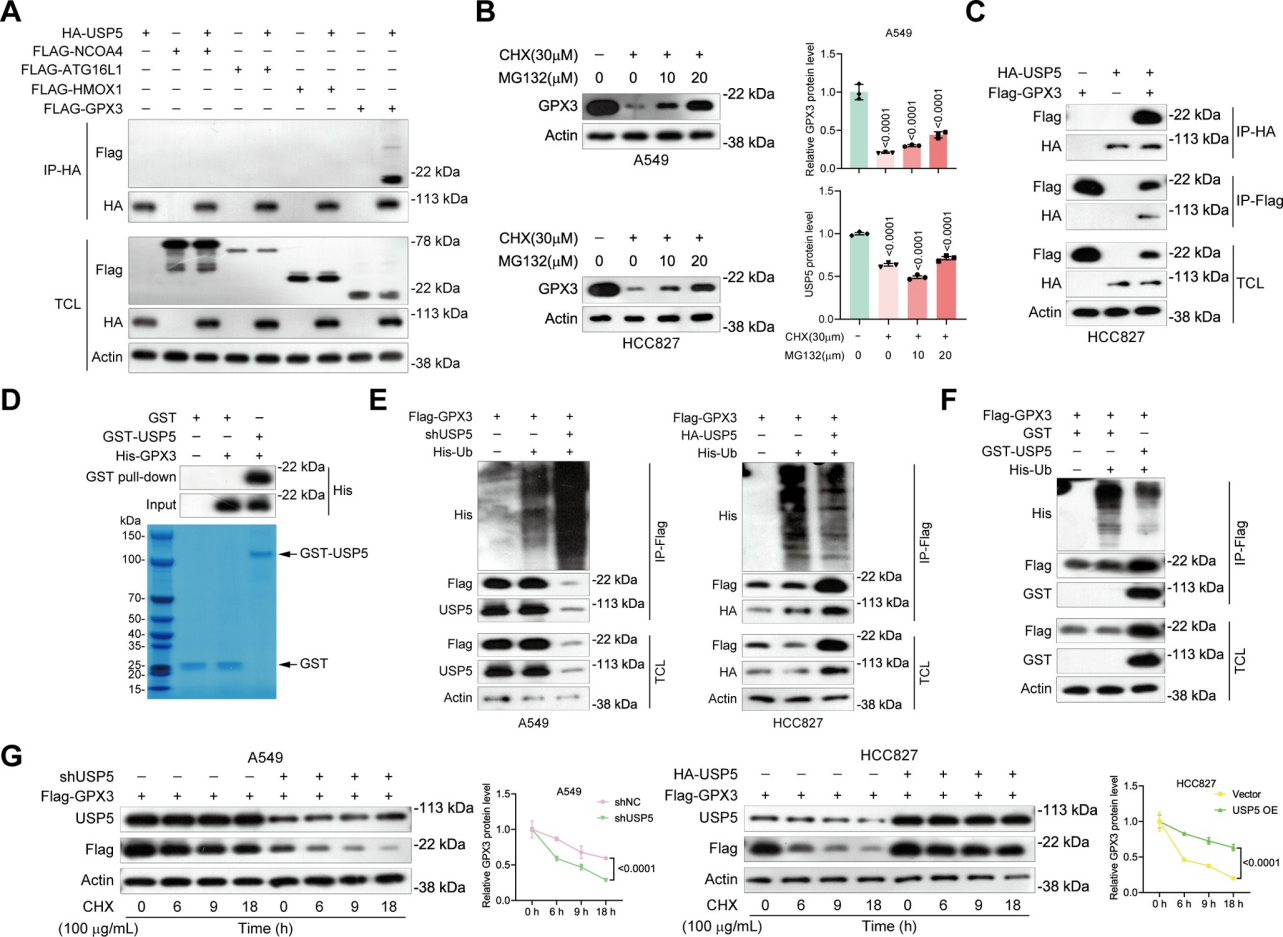
USP5 interacts with and stabilizes GPX3 through deubiquitination. (A) Co‐IP confirming the interaction between USP5 and GPX3 in A549 cells. (B) Western blot showing GPX3 degradation following CHX treatment and protection by MG132 in LUAD cells. (C) Co‐IP showing the interaction between USP5 and GPX3 in HCC827 cells. (D) GST pull‐down assays validating direct binding between USP5 and GPX3. (E, F) Ubiquitination assays showing increased GPX3 ubiquitination upon USP5 depletion and decreased ubiquitination upon overexpression (E); in vitro assays confirming this effect using GST‐USP5 (F). (G) CHX chase assay indicating GPX3 stabilization by USP5 in LUAD cells. TCL, total cell lysate. Statistical analyses were conducted using two‐way ANOVA for (G).

### 3.4 GPX3 mediates USP5's anti‐ferroptotic function in LUAD cells

To validate the functional relevance of GPX3 in USP5‐mediated ferroptosis suppression, we overexpressed GPX3 in USP5‐deficient A549 cells and silenced it in USP5‐overexpressing HCC827 cells in the presence of erastin. Knockdown and overexpression efficiency were confirmed by qPCR and Western blotting (Supporting Information S1: Figure S4A,B). MTT and colony formation assays demonstrated that GPX3 overexpression improved viability and colony‐forming ability in USP5‐deficient A549 cells, whereas GPX3 silencing impaired the protective effects of USP5 in HCC827 cells (Figure 5A,B). Additionally, GPX3 overexpression facilitated the cell cycle progression and enhanced the migration and invasion capabilities of USP5‐deficient A549 cells. By contrast, GPX3 silencing functioned oppositely in USP5‐overexpressing HCC827 cells, as evidenced by 7‐AAD staining and Transwell assay data (Figure 5C–E).

**FIGURE 5:**
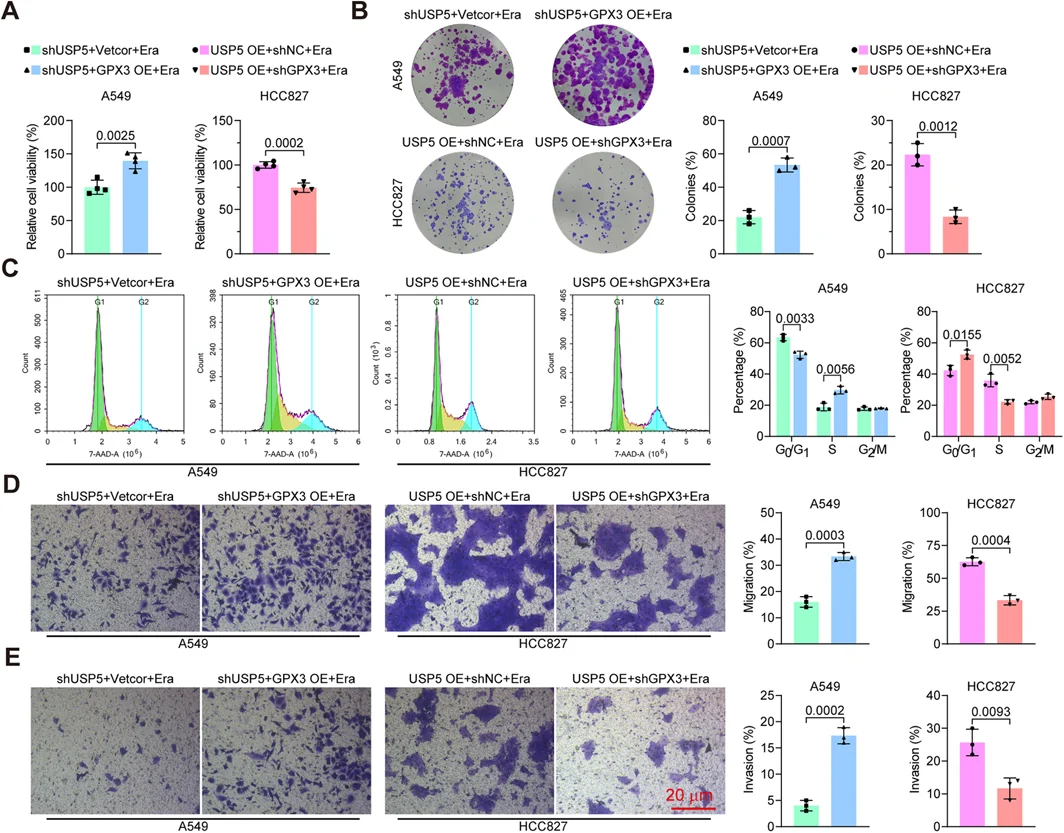
GPX3 mediates USP5‐driven malignant transformation in LUAD cells. (A) MTT assays showing that GPX3 overexpression improves, and knockdown exacerbates, viability defects in USP5‐deficient or overexpressing LUAD cells exposed to erastin. (B) Colony formation assays showing similar rescue or suppression patterns. (C) Cell cycle assays confirmed that GPX3 modulates cell cycle progression in LUAC cells with differential USP5 expression. (D, E) Transwell assays showing GPX3 rescues impaired migration/invasion caused by USP5 deficiency, whereas GPX3 depletion mitigated the effect of USP5 overexpression. Statistical significance was determined by unpaired bilateral Student's *t*‐tests.

To directly assess the impact of GPX3 on the regulation of ferroptosis by USP5 in erastin‐treated LUAD cells, we first performed biochemical assays. The results showed that GPX3 overexpression decreased the levels of MDA, GSSG, and Fe^2+^, while increasing GSH levels in USP5‐deficient A549 cells. Conversely, GPX3 knockdown resulted in elevated levels of MDA, GSSG, and Fe^2+^, along with a notable reduction in GSH concentration in USP5‐overexpressing HCC827 cells (Figure 6A–E). Moreover, DCF‐DA staining showed that ROS accumulation was diminished in USP5‐deficient A549 cells upon GPX3 overexpression, whereas depletion of GPX3 in USP5‐overexpressing HCC827 cells enhanced ROS levels (Figure 6F). BODIPY 581/591 C11 staining demonstrated that GPX3 overexpression reduced lipid peroxidation in USP5‐deficient A549 cells, whereas GPX3 deletion elevated lipid peroxidation in USP5‐overexpressing HCC827 cells (Figure 6G). Additionally, we examined the mitochondrial structure using TEM, demonstrating that GPX3 overexpression improved mitochondrial morphology and preserved structural integrity in USP5‐deficient A549 cells. In contrast, GPX3 knockdown in USP5‐overexpressing HCC827 cells exacerbated mitochondrial damage (Figure 6H). At the molecular level, Western blot analysis revealed that GPX3 overexpression significantly increased the expression of SLC7A11, GPX4, and FTH1 in USP5‐deficient A549 cells, whereas GPX3 depletion reduced the expression of these ferroptosis repressors in USP5‐overexpressing HCC827 cells (Figure 6I). Taken together, these findings strongly support the conclusion that GPX3 is a critical mediator of USP5's ability to suppress ferroptosis in LUAD cells.

**FIGURE 6:**
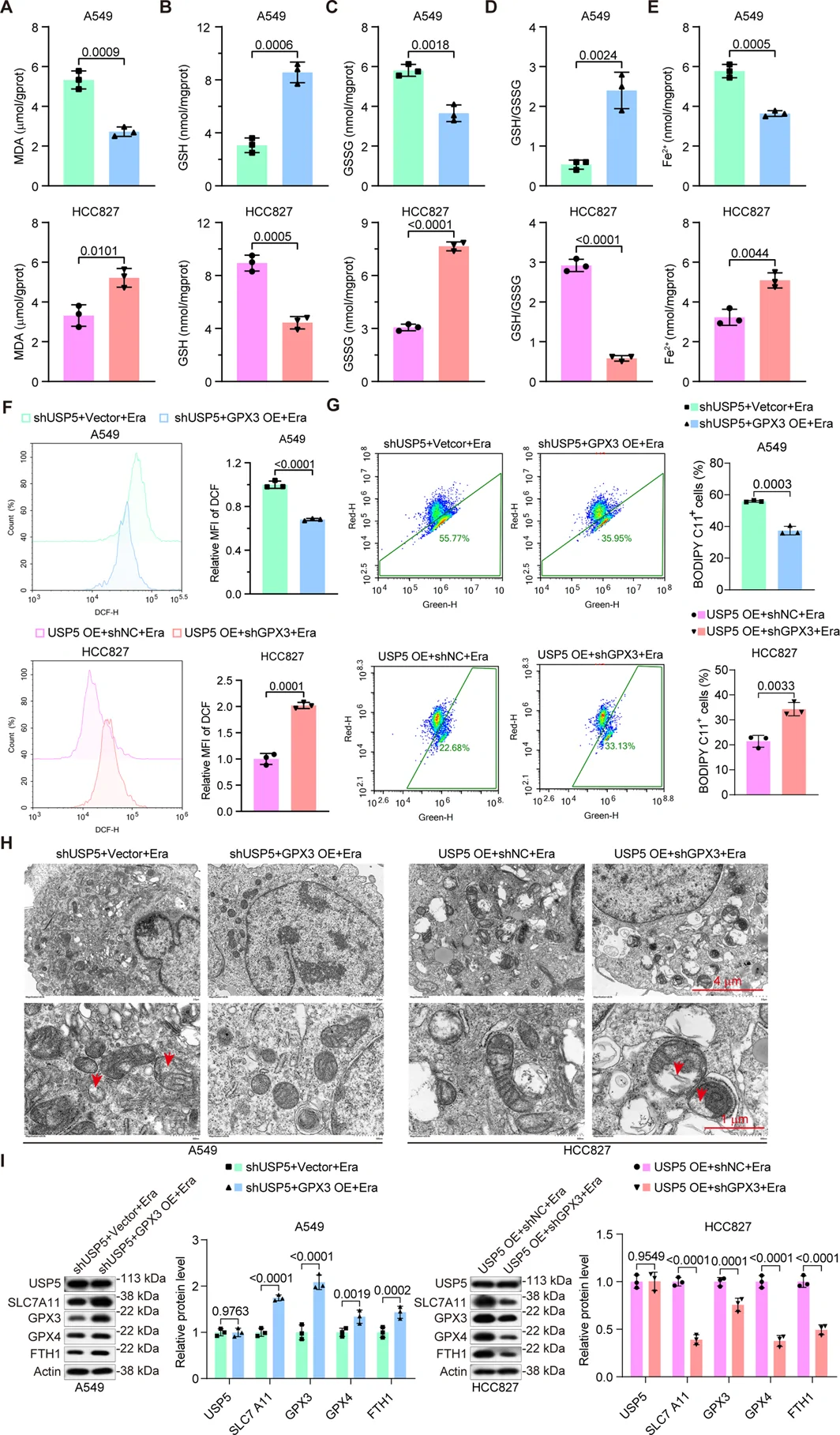
GPX3 regulates ferroptosis markers and mitochondrial integrity downstream of USP5. (A‐E) Biochemical analyses of MDA, GSH, GSSG, and Fe^2+^ were performed on cells in which GPX3 was knocked down in the presence of USP5 overexpression, or in which GPX3 was overexpressed in the presence of USP5 knockdown. (F) DCF‐DA staining showing ROS modulation by GPX3 in USP5‐deficient or overexpressing LUAD cells. (G) BODIPY 581/591 C11 staining confirmed GPX3‐mediated regulation of intracellular lipid peroxidation in USP5‐modified LUAD cells. (H) TEM images displaying mitochondrial restoration by GPX3 overexpression and silencing in USP5‐deficient or overexpressing LUAD cells. Red arrows indicated damaged mitochondria. (I) Western blotting of ferroptosis suppressors (SLC7A11, GPX4, and FTH1) in response to GPX3 modulation under USP5 perturbation. *p*‐values were determined by unpaired bilateral Student's *t*‐tests. ROS, reactive oxygen species.

### 3.5 USP5 deficiency inhibits growth and suppresses ferroptosis in LUAD organoids derived from patient tumors

To further explore the functional role of USP5 in LUAD, we established LUAD organoid lines derived from primary tumor tissues of three LUAD patients. These organoids displayed typical three‐dimensional morphology and organization (Supporting Information S1: Figure S5A,B) and expressed established LUAD markers, including CK7, Napsin A, and TTF1,^25^, ^26^ along with proliferation marker Ki‐67 (Supporting Information S1: Figure S5C–F), indicating the successful establishment of LUAD organoids. To induce ferroptosis, the organoids were exposed to various concentrations of erastin, and Fe^2+^ level assays identified 80 μM as the optimal working concentration for subsequent experiments (Supporting Information S1: Figure S5G). Next, we depleted USP5 in these organoids and exposed them to erastin. H&E staining showed that neither erastin treatment nor USP5 knockdown led to overt structural changes in organoid architecture (Figure 7A). However, IF analysis revealed that erastin markedly reduced cell proliferation, an effect that was further potentiated by USP5 deficiency, as evidenced by a decrease in Ki‐67‐positive cells (Figure 7B,E). In line with this observation, IF staining also showed that the expression of LUAD identity markers, such as CK7 and Napsin A, was diminished following erastin treatment and further reduced by USP5 knockdown (Figure 7C,D,F,G), indicating compromised LUAD characteristics. Morphologically, USP5‐deficient organoids displayed a significantly attenuated size increase following erastin exposure, in comparison to the control group (Figure 7H).

**FIGURE 7:**
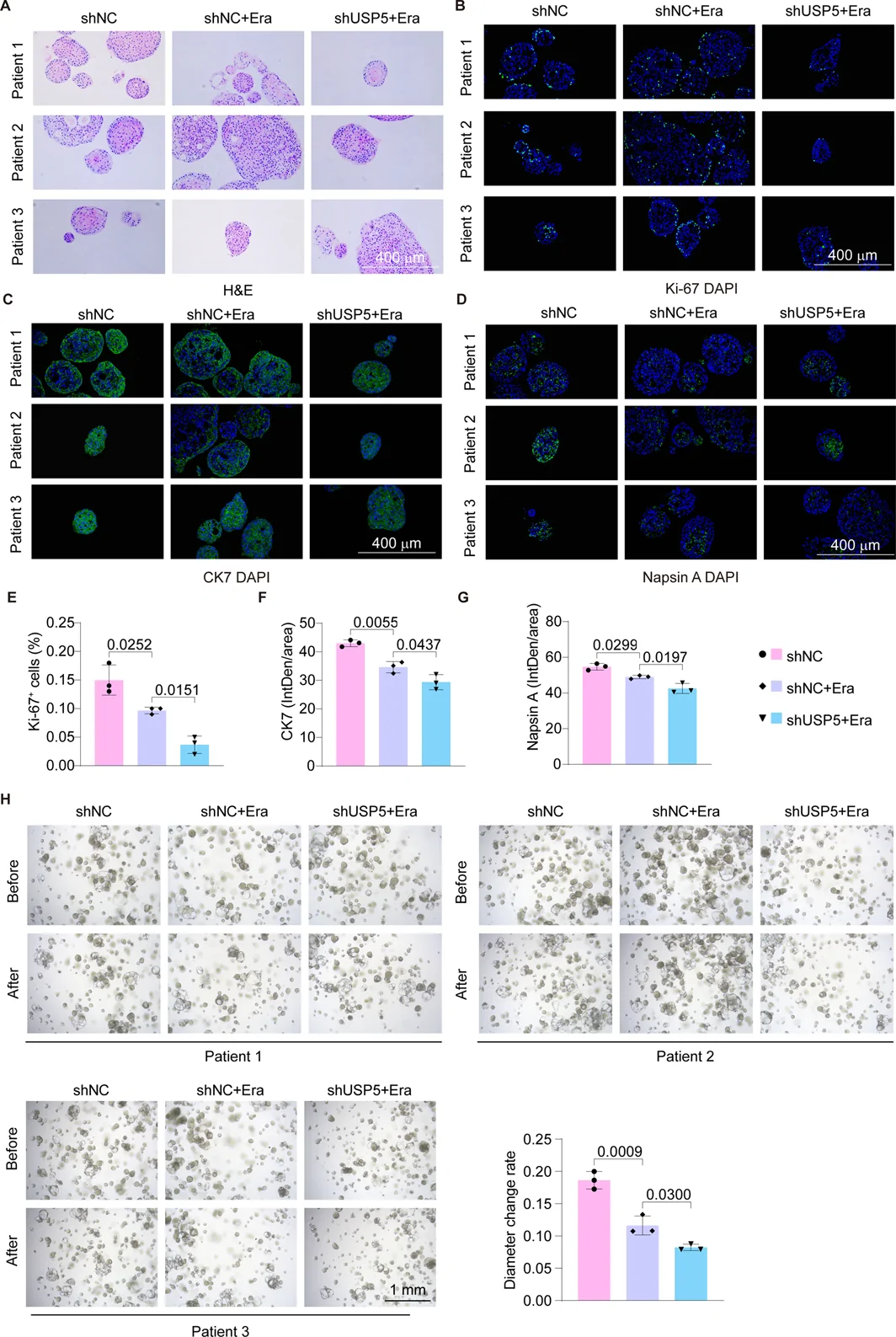
USP5 depletion enhances erastin‐mediated growth inhibition and loss of LUAD markers in organoid models. (A) H&E staining showing preserved morphology following USP5 depletion and erastin treatment. (B–G) Immunofluorescence for Ki‐67 (B, E), CK7 (C, F), and Napsin A (D, G) showing reduced proliferation and LUAD identity upon erastin treatment with/without USP5 depletion. (H) Morphology and quantification of organoid diameters during a 5‐day period under different conditions. *p*‐values were determined by one‐way ANOVA followed by Tukey's HSD tests.

Biochemical assays further showed that erastin treatment elevated the levels of MDA, GSSG, and Fe^2+^, while depleting GSH; these changes were all exacerbated by USP5 knockdown (Figure 8A–E). TEM revealed that USP5 loss intensified erastin‐induced mitochondrial damage in the organoids, further highlighting the role of USP5 in preserving mitochondrial integrity under ferroptotic stress (Figure 8F). Molecularly, Western blot analysis showed that erastin reduced the expression of USP5, GPX3, GPX4, and FTH1 while upregulating the ferroptosis marker COX2.^27^ USP5 depletion amplified all these erastin‐induced changes (Figure 8G). Taken together, these findings emphasize the crucial function of USP5 in protecting LUAD organoids against ferroptosis and maintaining their proliferative and molecular characteristics.

**FIGURE 8:**
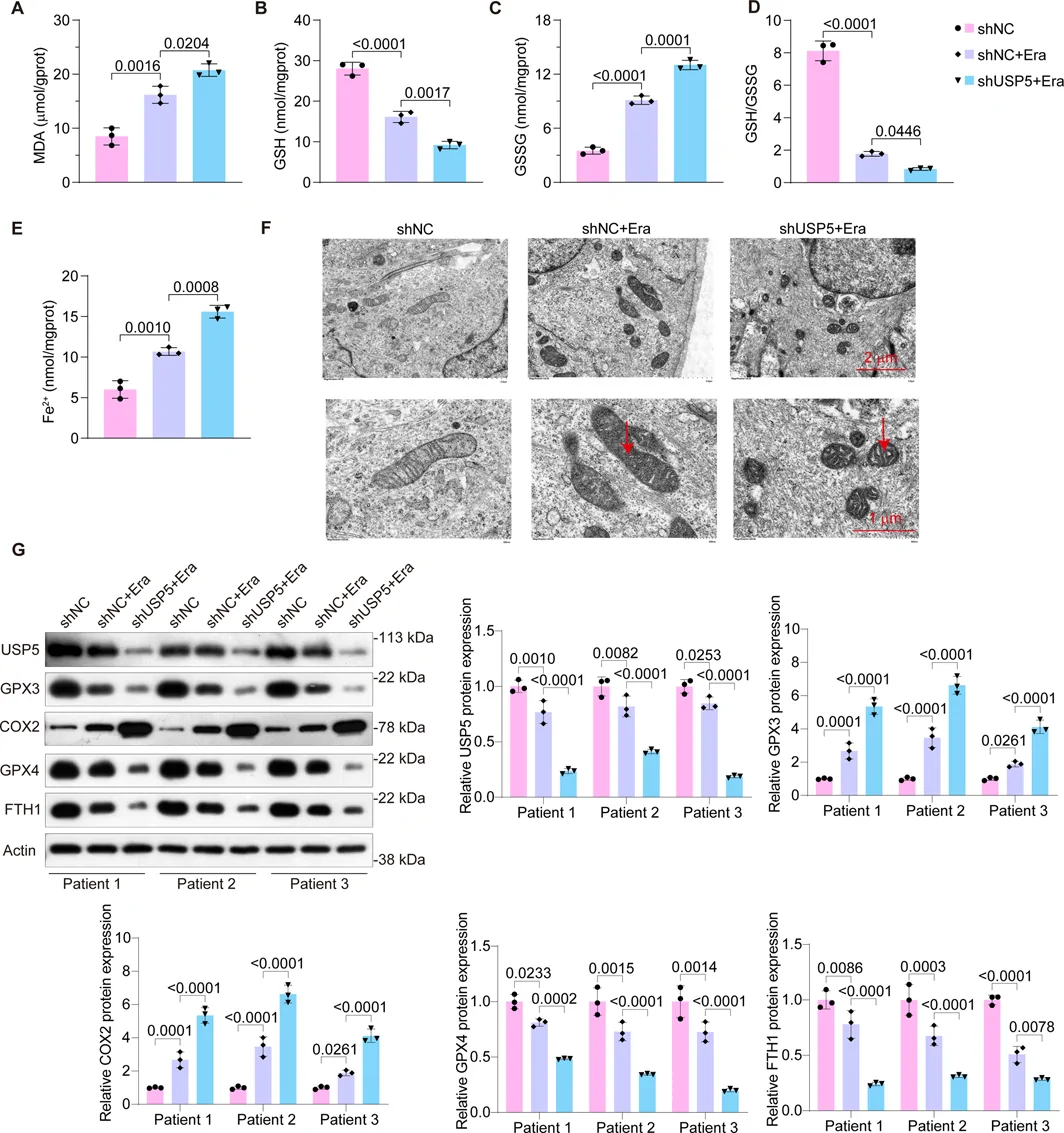
USP5 inhibits ferroptosis in patient‐derived LUAD organoids with erastin treatment. (A‐E) Biochemical assays showing altered MDA, GSH, GSSG, and Fe^2+^ levels in USP5‐depleted organoids with erastin treatment. (F) TEM images showing mitochondrial damage in erastin‐treated control and USP5‐deficient organoids. (G) Western blots showing reduced expression of USP5, GPX3, GPX4, and FTH1 and increased COX2 levels upon USP5 depletion in organoids treated with erastin. *p*‐values were determined by one‐way ANOVA followed by Tukey's HSD tests.

## 4 DISCUSSION

In this study, we identify a previously unrecognized role for USP5 in promoting LUAD progression through the suppression of ferroptosis. Our findings demonstrate that USP5 directly interacts with and stabilizes GPX3, a known antioxidant enzyme, by reducing its ubiquitination, thereby enhancing its capacity to counteract ferroptotic stress. This mechanism appears critical for maintaining LUAD cell survival and aggressiveness, both in established cell lines and in patient‐derived organoids.

Previous studies have reported the oncogenic role of USP5 in various cancers through distinct mechanisms. In NSCLC, USP5 has been shown to drive tumorigenesis by stabilizing β‐catenin, cyclin D1, and PD‐L1.^28^, ^29^, ^30^, ^31^ In other cancer types, USP5 facilitates pancreatic cancer progression by enhancing the stability of FoxM1,^32^ is overexpressed in bladder cancer, where it promotes tumorigenesis via c‐Jun stabilization,^33^ and enhances colorectal cancer growth by stabilizing TUFM.^34^ Furthermore, there have been studies on the role of USP5 in ferroptosis. In colorectal cancer (CRC), USP5 promotes the degradation of YBX3 (Y‐box‐binding protein 3), stabilizes SLC7A11, and inhibits ferroptosis, thereby promoting the survival and tumor progression of colorectal cancer cells.^35^ In a further two studies, it was found that USP5 stabilizes GPX4, thereby inhibiting ferroptosis in bladder and breast cancer cells.^36^, ^37^ Consistent with previous studies, we have demonstrated that GPX3 acts as a novel substrate for USP5, reducing its ubiquitination and stabilizing GPX protein expression, thereby exerting an anti‐ferroptotic effect in LUAD. This mechanism supports a new oncogenic dimension of USP5 in LUAD, one that is centered on protecting cells from ferroptotic damage. Given the broad range of proteins regulated by USP5, it is possible that additional downstream targets contribute to its anti‐ferroptotic effects, which will require further investigation. Moreover, whether USP5 exerts a similar anti‐ferroptotic role in LUSC remains to be determined.

The mechanism driving USP5 overexpression in NSCLC remains partially understood. Although previous reports have suggested that genetic amplification and reduced DNA methylation may contribute to increased USP5 expression,^38^ post‐transcriptional regulatory mechanisms may also be involved. For example, miRNAs and circular RNAs have been implicated in regulating USP5 expression in other contexts.^39^, ^40^ Whether similar post‐transcriptional mechanisms operate in NSCLC needs further exploration. Interestingly, our data show that erastin treatment does not affect USP5 mRNA levels but leads to reduced USP5 protein levels in A549 cells, suggesting regulation at the post‐translational level. However, this effect was not observed in HCC827 cells, indicating that erastin's influence on USP5 may be cell‐context‐dependent. Notably, similar suppression of USP5 protein by erastin was observed in LUAD organoids, suggesting that this effect may be relevant in clinical tumor settings as well.

GPX3, the substrate of USP5 identified in this study, is a conserved glutathione peroxidase family member and an important ROS scavenger in extracellular and plasma compartments. Like GPX4, GPX3 is capable of neutralizing lipid hydroperoxides, which are key drivers of ferroptosis.^41^, ^42^ Although GPX3 is generally regarded as a tumor suppressor, its function appears to vary based on the cellular and pathological context.^42^ In lung cancer, serum GPX3 levels have been linked to disease recurrence after surgery.^43^ Meanwhile, its tumor‐suppressive role has been attributed to redox regulation and downstream signaling modulation.^44^, ^45^ Our results indicate that in the context of ferroptotic stress in LUAD, GPX3 acts as an anti‐ferroptotic factor that supports tumor cell viability. Overexpression of GPX3 in USP5‐deficient LUAD cells restored cell survival, reduced ferroptotic indicators such as lipid peroxidation and ROS levels, improved the mitochondrial structure, and promoted colony formation and invasion. Conversely, the knockdown of GPX3 in USP5‐overexpressing LUAD cells reversed these effects and increased ferroptotic damage. These findings establish GPX3 as an essential effector of USP5's anti‐ferroptotic function in LUAD and suggest that, in certain pathological settings, GPX3 may function in a tumor‐promoting capacity.

In terms of regulation, GPX3 expression in NSCLC cells has been shown to be controlled by noncoding RNAs.^45^ In other cancer contexts, the E3 ubiquitin ligase TRIM6 has been implicated in facilitating GPX3 degradation via ubiquitination.^46^ Whether TRIM6 or other E3 ligases mediate GPX3 ubiquitination in LUAD remains unknown. Additionally, the other upstream regulators that determine GPX3 stability and activity in the ferroptosis response require further clarification. Understanding the broader regulatory network controlling GPX3 will help define how LUAD cells adapt to oxidative stress and resist ferroptosis.

Despite the comprehensive in vitro and organoid‐based investigations presented in our study, several limitations should be acknowledged. First, we did not include in vivo validation using xenograft or genetically engineered mouse models, which would provide critical insights into the physiological relevance of USP5 in regulating ferroptosis and LUAD progression within the tumor microenvironment. Second, although we demonstrated the anti‐ferroptotic role of USP5 in patient‐derived LUAD organoids, we did not perform rescue experiments in this system. Third, although we identified a direct interaction between USP5 and GPX3 and demonstrated USP5‐dependent deubiquitination of GPX3, the specific domains mediating this interaction remain undefined. Further structural or mutagenesis studies are needed to pinpoint the binding interfaces between USP5 and GPX3. Additionally, we have not characterized the type of ubiquitin linkage (e.g., K48‐, K63‐, or other atypical linkages) removed by USP5 on GPX3, which is critical for understanding the precise regulatory mechanism. Lastly, although our analysis of TCGA and GEO datasets suggests a prognostic role of USP5 in LUAD, these findings are based on limited sample sizes. Validation in larger, independent patient cohorts with detailed clinical annotations is necessary to confirm the prognostic utility of USP5 expression and its potential as a biomarker for patient stratification in ferroptosis‐targeted therapies.

In summary, our study identifies USP5 as a key negative regulator of ferroptosis in LUAD through stabilization of GPX3. USP5 directly binds to GPX3 and removes its ubiquitin chains, thereby preventing its proteasomal degradation and enhancing its antioxidant function. This interaction promotes LUAD cell survival, proliferation, and invasion under ferroptotic stress, both in traditional cell models and in patient‐derived organoids. Our findings reveal a novel USP5–GPX3 axis that contributes to ferroptosis resistance and tumor progression in LUAD. Targeting this pathway may offer a new strategy for enhancing ferroptosis‐based therapies in LUAD.

## AUTHOR CONTRIBUTIONS

**Xin Fan**: Conceptualization; funding acquisition; methodology; resources; project administration; writing—original draft. **Yubiao Lin**: Conceptualization; funding acquisition; supervision; writing—review and editing. **Zhigao Zhen**: Data curation; formal analysis; investigation; methodology; writing—review and editing. **Yingqin Gao**: Formal Analysis; software; validation; visualization; writing—review and editing. **Wenhui Zhen**: Data curation; formal analysis; validation; writing—review and editing. **Siyuan Zeng**: Resources; visualization; writing—review and editing. **Yufei Ji**: Resources; visualization; writing—review and editing. **Kaida Huang**: Investigation; methodology; resources; funding acquisition; writing—review and editing. **Shuitu Feng**: Conceptualization; funding acquisition; supervision; writing—review and editing.

## CONFLICT OF INTEREST STATEMENT

The authors declare no conflicts of interest.

## ETHICS STATEMENT

This study was approved by the Medical Ethics Committee of Xiamen Haicang Hospital (NO. KY‐2022006) in September 2022. We adhere to the ethical standards for medical research involving human subjects as set out in the Declaration of Helsinki. All participants provided written informed consent prior to joining the study.

## Supporting information

Supporting Information S1

Table S3

## Data Availability

The data that support the findings of this study are available from the corresponding author upon reasonable request.
