# Supplementary material for: Deubiquitinase USP5 suppresses ferroptosis in lung adenocarcinoma by deubiquitinating and stabilizing GPX3
Source: J Cell Commun Signal. 2026 Oct 24;20(4):e70121. doi: 10.1002/ccs3.70121 (PMC13695517; doi:10.1002/ccs3.70121)
Supplement: Supplementary file 1 [file CCS3-20-e70121-s001.docx]

**Supplementary Table**

Supplementary Table 1. Primers for plasmid construction.

| **Plasmid** | **Sequence (5' to 3')** |
| --- | --- |
| pLKO.1-shUSP5-1 | CCGGCCTGTCTGTAAGGAGACTTTGCTCGAGCAAAGTCTCCTTACAGACAGGTTTTT |
|  | AATTAAAAACCTGTCTGTAAGGAGACTTTGCTCGAGCAAAGTCTCCTTACAGACAGG |
| pLKO.1-shUSP5-2 | CCGGGACCACACGATTTGCCTCATTCTCGAGAATGAGGCAAATCGTGTGGTCTTTTT |
|  | AATTAAAAAGACCACACGATTTGCCTCATTCTCGAGAATGAGGCAAATCGTGTGGTC |
| pLKO.1-shUSP5-3 | CCGGGATAGACATGAACCAGCGGATCTCGAGATCCGCTGGTTCATGTCTATCTTTTT |
|  | AATTAAAAAGATAGACATGAACCAGCGGATCTCGAGATCCGCTGGTTCATGTCTATC |
| pLVX-CMV-HA-USP5-pGK-puro | actcagatctcgaggccaccatggcggagctgagtgaggag |
|  | CGTAATCTGGTACGTCGTATGGGTAgctggccactctctggtagaag |
| pLVX-CMV-HA-USP14-pGK-puro | GGACTCAGATCTCGAGGCCACCATGCCGCTCTACTCCGTTAC |
|  | AATCTGGTACGTCGTATGGGTACTGTTCACTTTCCTCTTC |
| pLVX-CMV-HA-USP18-pGK-puro | GGACTCAGATCTCGAGGCCACCATGAGCAAGGCGTTTGGGCTC |
|  | AATCTGGTACGTCGTATGGGTAGCACTCCATCTTCATGTAAACC |
| pLVX-CMV-HA-USP39-pGK-puro | GGACTCAGATCTCGAGGCCACCATGTCCGGCCGGTCTAAGCG |
|  | CTGGTACGTCGTATGGGTAAGCCCCCTGCTGGTTGGTTTCATC |
| pcDNA3.1-FLAG-NCOA4 | TACCGAGCTCGGATCCGCCACCATGGGGGCCCAGTTGACCAC |
|  | GCCCTCTAGACTCGAGGAATCCACATGGGATCTGAAAATTC |
| pcDNA3.1-FLAG-ATG16L1 | TACCGAGCTCGGATCCGCCACCatgtcgtcgggcctccgcgc |
|  | GCCCTCTAGACTCGAGgtactgtgcccacagcacag |
| pcDNA3.1-FLAG-HMOX1 | TACCGAGCTCGGATCCGCCACCatggagcgtccgcaacccgac |
|  | GCCCTCTAGACTCGAGcatggcataaagccctacag |
| pcDNA3.1-FLAG-GPX3 | TACCGAGCTCGGATCCGCCACCATGGCCCGGCTGCTGCAGGCGTCC |
|  | GCCCTCTAGACTCGAGCTTCCTCTTGACCCCCAGG |
| pcDNA3.1-His-Ub | TAGAGAATTCGGATCCGCCACCatgcagatcttcgtgaagac |
|  | TGGTGATGGTGATGATGaccacctctgagacggaggac |
|  | CATAAGGGTAAAGCTTTCAATGGTGATGGTGATGATG |
| pLKO.1-shGPX3-1 | CCGGTACGGAGCCCTCACCATTGATCTCGAGATCAATGGTGAGGGCTCCGTATTTTT |
|  | AATTAAAAATACGGAGCCCTCACCATTGATCTCGAGATCAATGGTGAGGGCTCCGTA |
| pLKO.1-shGPX3-2 | CCGGCACCATTCGGTCTGGTCATTCCTCGAGGAATGACCAGACCGAATGGTGTTTTT |
|  | AATTAAAAACACCATTCGGTCTGGTCATTCCTCGAGGAATGACCAGACCGAATGGTG |
| pLKO.1-shGPX3-3 | CCGGGTGGAGGCTTTGTCCCTAATTCTCGAGAATTAGGGACAAAGCCTCCACTTTTT |
|  | AATTAAAAAGTGGAGGCTTTGTCCCTAATTCTCGAGAATTAGGGACAAAGCCTCCAC |
| shNC | CCGGTTCTCCGAACGTGTCACGTTTCTCGAGAAACGTGACACGTTCGGAGAATTTTT |
|  | AATTAAAAATTCTCCGAACGTGTCACGTTTCTCGAGAAACGTGACACGTTCGGAGAA |

Supplementary Table 2. Western blot antibody details

| **Target** | **Manufacturer** | **Catalog number** | **Dilution** |
| --- | --- | --- | --- |
| USP5 | Proteintech | 10473-1-AP | 1:500 |
| FTH1 | Abcam | ab75973 | 1:500 |
| SLC7A11 | ABclonal | A2413 | 1:500 |
| GPX4 | Proteintech | 67763-1-lg | 1:500 |
| GPX3 | ABclonal | A12956 | 1:500 |
| COX2 | Proteintech | 27308-1-AP | 1:500 |
| Actin | Proteintech | 20536-1-AP | 1:2000 |
| HA-tag | Proteintech | 51064-2-AP | 1:2000 |
| Flag-tag | Proteintech | 20543-1-AP | 1:2000 |
| His-tag | Proteintech | 66005-1-Ig | 1:2000 |
| HRP-Anti-mouse IgG | Proteintech | SA00001-1 | 1:5000 |
| HRP-Anti-Rabbit IgG | Proteintech | SA00001-2 | 1:5000 |

**Supplementary Figure**

**Figure S1. Expression patterns of candidate USPs in NSCLC patient subgroups.**
(A-E) Boxplots illustrating USP5 (A), USP14 (B), USP18 (C), USP28 (D), and USP39 (E) expression across different age groups, sexes, survival statuses (alive vs. deceased), and tumor stages in the TCGA-LUNG dataset. *, P<0.05; **, P<0.01; ***, P<0.001; ns, not significant.

**Figure S2. Functional enrichment and gene correlation analyses associated with USP5.**
(A) GO analysis (including BP, CC, and MF) and KEGG pathway enrichment analysis of USP5-associated differentially expressed genes in LUAD, indicating enrichment in cell division, DNA replication, and mitotic cell cycle regulation pathways. (B) Pearson correlation analysis showing positive associations between USP5 mRNA levels and known ferroptosis-regulating genes (GPX4, NRF2, RGS4, SLC7A11) in LUAD patients from the TCGA-LUNG dataset.

**Figure S3. Construction of an ferroptosis cell model and detection of USP5 expression.**

(A) CCK8 assay data showing IC_50_ of erastin in A549 (20.4 μM) and HCC827 (36 μM) cells. (B) MTT assay comparing the viability of LUAD cells overexpressing different USPs (USP5, USP14, USP18, USP39) after erastin treatment, highlighting a protective effect of USP5. (C) qPCR validation of three USP5 shRNAs in HEK293T cells. (D) Confirmation of USP5 knockdown and overexpression by qPCR in A549 and HCC827 cells, with or without erastin treatment.

**Figure S4. Validation of GPX3 knockdown and overexpression in LUAD cells.**
(A) qPCR data showing the mRNA levels of GPX3 in HEK293T cells transfected with shGPX3s. P-values were determined by One-way ANOVA followed by Tukey's HSD tests. (B) qPCR data showing the mRNA levels of GPX3 in HEK293T cells transfected with GPX3 OE. P-values were calculated by unpaired bilateral Student’s t-tests.

**Figure S5. Characterization of patient-derived LUAD organoids and erastin response.**
(A, B) Bright-field microscopy images showing morphological features of LUAD organoids from three patients. (C-F) IF staining showing positive expression of LUAD markers CK7 (C), Ki-67 (D), Napsin A (E), and TTF1 (F) in the organoids. (G) Fe^2+^ level assay establishing 80 μM erastin as the effective concentration for inducing ferroptosis in organoids. One-way ANOVA followed by Tukey's HSD tests were used to calculate the P-values.
